# News and Notes

**Published:** 1899-06

**Authors:** 


### *NEWS AND NOTES.*

THE Collier Bill prohibiting the New York Board of Health from selling antitoxin and vaccine has been defeated.

THE brain of the late Professor Helmholtz weighed forty-five ounces, and the convolutions were exceedingly complex.

DR. MOSES D. HOGE, JR., of Richmond, Va., has recently been elected a Fellow of the Royal Microscopical Society of London.

THE *Kansas City Medical Index* and the *Kansas City Lancet* have consolidated. Dr. John Punton is the editor, with numerous collaborators.

THE next President of the British Medical Association is to be Sir Wm. Turner, M.D., F.R.S., Professor Anatomy in the University of Edinboro.

DR. A. J. C. SKENE, who recently resigned from the faculty of the Long Island Hospital College, has been succeeded as professor of gynecology by Dr. Michalson.

SIXTY thousand marks ($15,000) have been appropriated by the German Reichstag to defray the approaching Koch expedition to the tropics to investigate the origin and nature of malaria.

THE trustees of St. Luke's Hospital, New York, have passed resolutions showing their appreciation of the heroism displayed by Miss Frances Troup, a graduate of St. Luke's Training School for Nurses, in saving the life of her patient at the burning of the Windsor Hotel.

PHOTOGRAPHING THE STOMACH.—Drs. Lang and Meltzing of Berlin, have invented an apparatus for photographing the interior of the stomach. A very small camera and an electric light are carried into the stomach in a tube, the stomach is then emptied, filled with air, and the photograph made. The negative is about the size of a cherry-stone, but can be enlarged.

---

CHATTANOOGA MEDICAL COLLEGE.—The tenth commencement exercises of this school took place on March 21st, and was a very creditable affair. Dean Cobleigh stated that the session just ended marked the close of the first decade and was the most prosperous in the college history, that nearly two hundred students had been enrolled in the college class during the past term, and that, with the completion of the new city hospital, the outlook of the institution was particularly promising.

---

ANNOUNCEMENT.—Congrès périodique international de Gynécologie et d'Obstétrique, 3e Section, Amsterdam, Aout, 1899. Secrétariat, Sarphatistraat 1h.—Amsterdam, September, 1898. Dear Sir: We have the honor of soliciting your presence at the 3d Intern. Congress for Gynecology and Obstetrics, to take place at Amsterdam from the 8th to the 12th of August, 1899, under the patronage of the Minister of the Interior. The leading questions for discussion will be the following: 1. The surgical treatment of fibro-myoma. 2. The relative value of antiseptics and improved technic for the actual results in Gynecological Surgery. 3. The influence of posture on the form and dimensions of the pelvis. 4. The indication for Cæsarean section compared to that for symphyseotomy, craniotomy and premature induction of labor. We have succeeded in obtaining the valuable concurrence as reporters of MM. Doyen, Howard Kelly and Schauta, who will treat the first question; MM. Bumm, Richelot and Lawson Tait the second; MM. Bonnaire, Pinzani and Walcher the third, and MM. Leopold, Pinard, Pestalozza and Fancourt Barnes the fourth. We propose sending the reports, with their translations in the official languages, to all the members a month before the opening of the Congress. As regards private communications, preference will be given to those bearing upon the above mentioned leading questions. Time will also be allowed sufficient for any demonstrations kindly afforded by the members. The official languages are English, French, German and Italian. We venture to urge our request that you will honor the Congress with your presence, and by communicating your experience insure scientific results as satisfactory as those obtained by the previous Congresses of Brussels and Geneve. Subscription Form— I (name, surname and quality), residing at (exact address), hereby declare my adherence to the 3d Session of the periodical International Congress for Gynecology and Obstetrics, to be held at Amsterdam on August 8th, 1899, and agree to pay the sum of One Guinea for my share of the contribution. Signature. I------ ------founder (*membre fondateur*) (those who having paid the sum of 300 francs are thereby exempt from all further contribution to future congress) of the International Congress for Gynecology and Obstetrics, hereby state my intention to be present at the next Congress, which will take place at Amsterdam, August 8th, 1899. Signature. Please address all communications to J. D. Emmet, M.D., Secretary for America, 91 Madison Avenue, New York, N. Y. The Committee: H. Treub, president; J. Veit, vice-president; G. C. Nijhoff, J. P. Barnouw, treasurer; M. A. Mendes de Leon, secretary.

